# Stereotactic body radiation therapy for oligometastatic melanoma: a real-world study of the ESTRO/EORTC nomenclature

**DOI:** 10.1186/s13014-026-02792-2

**Published:** 2026-01-30

**Authors:** Ellen Heurlin, Vitali Grozman, Suzanne Egyhazi Brage, Karin Lindberg, Hildur Helgadottir

**Affiliations:** 1 Department of Oncology-Pathology, Karolinska Institutet, Stockholm, Sweden; 2 Department of Diagnostic Radiology, Karolinska University Hospital, Stockholm, Sweden; 3 Department of Molecular Medicine and Surgery, Karolinska Institutet, Stockholm, Sweden; 4 Department of Oncology, Karolinska University Hospital, Stockholm, Sweden

**Keywords:** Melanoma, Stereotactic body radiation therapy, SBRT, Oligometastatic

## Abstract

**Background:**

The study aims to investigate the efficacy of stereotactic body radiation therapy (SBRT) in melanoma patients with oligometastatic disease (OMD), and to assess the prognostic value of the European Society for Radiotherapy and Oncology (ESTRO) and European Organization for Research and Treatment of Cancer (EORTC) nomenclature for these patients.

**Method:**

This is a single-center, retrospective study including all melanoma patients with OMD (*n* = 66) receiving SBRT between 2010 and 2023. Patients were categorized based on the timing of SBRT of OMD according to the ESTRO/EORTC classification. We analyzed local control, progression-free survival (PFS), overall survival (OS), safety, and prognostic factors.

**Results:**

The median follow-up was 72.5 months. Patients were categorized at the timepoint of SBRT as having de novo (*n* = 20), repeat (*n* = 25), or induced (*n* = 21) OMD. The most common OMD subcategories were repeat oligorecurrence (33.3%) and metachronous oligorecurrence (16.7%). Concurrent systemic treatment was administered in 30.3% of the patients. Local control rates at 1, 2, and 3 years was 96.3%, 93.2%, and 93,2%, respectively. The median PFS and OS were 7.7 (95% CI 4.9–12.4) and 26.5 (95% CI 17.6–38.8) months, respectively. No significant differences in PFS or OS were observed between patients with de novo, repeat, or induced OMDs. Similarly, survival outcomes did not differ between patients classified into the oligorecurrence, oligoprogression, or oligopersistence cohorts. However, patients who underwent SBRT targeting all metastatic sites demonstrated significantly improved PFS and OS compared to those with additional non-irradiated lesions (*p* = 0.022 and *p* = 0.002, respectively). Moreover, patients with a single metastasis had significantly better PFS and OS than those with 2–5 metastases (*p* = 0.045 and *p* = 0.021). However, only ECOG performance status remained significant in the multivariable analysis. Additionally, 19 patients (29%) experienced grade 1–2 SBRT-related side effects.

**Conclusion:**

SBRT was well tolerated and demonstrated excellent local control rates in melanoma with OMD. Our findings indicate that there was no significant difference in PFS or OS between the OMDs, suggesting that the prognostic implication of the ESTRO/EORTC classification in melanoma may warrant further evaluation in prospective studies.

**Supplementary Information:**

The online version contains supplementary material available at 10.1186/s13014-026-02792-2.

## Background

Until the early 2010s, metastatic melanoma had limited treatment options and a dismal prognosis with a median survival of only 6 months [1]. Significant improvements have occurred since then, primarily owing to the breakthrough of targeted therapies and immune checkpoint inhibitors (ICI) [2–4]. 

Despite these advances, survival outcomes for patients with metastatic melanoma vary widely. Several prognostic factors contribute to this variability, including Eastern Cooperative Oncology Group (ECOG) performance status, levels of serum albumin and lactate dehydrogenase (LDH) in the blood, as well as numbers and locations of metastases [5, 6]. Patients with metastatic cancers can be further categorized into those with limited number of lesions (oligometastatic) and those with widespread disease. The term oligometastatic disease (OMD) was first defined in 1995 by Hellman and Weichselbaum, which is described as a transitional state between localized and disseminated metastatic disease [7]. Although patients are categorized as having OMD, the definition has varied between studies, and demonstrated significant variation in survival outcomes [8].

With this background, the European Society for Radiotherapy and Oncology (ESTRO) and European Organization for Research and Treatment of Cancer (EORTC) established a classification system for OMD. The classification comprises three overarching categories: de novo (individuals experiencing OMD for the first time), repeat (those with a prior history of OMD), and induced (patients with a history of polymetastatic disease). These primary categories further diversify into nine distinct subcategories [9]. Five subsequent retrospective validation studies of the ESTRO/EORTC nomenclature revealed a difference in survival between the OMD categories treated with stereotactic body radiation therapy (SBRT). Two of these studies included mixed histologies, with lung cancer being predominant in the first study, while prostate, colorectal, and breast cancer were most common in the second study [10, 11]. The remaining three studies focused on specific histologies, with two exclusively investigating prostate cancer and one solely addressing non-small cell lung cancer (NSCLC) [12–14]. In contrast, a recent study, which is the only published study focusing on melanoma patients, did not identify any significant differences between the OMD groups when assessing local control, progression-free survival (PFS) or overall survival (OS) [15].

Additional studies, with limited representation of melanoma cases, indicate that patients with OMDs may derive benefit from the addition of local ablative treatment alongside systemic therapy [16–20]. This challenges the conventional idea that all metastatic cancer should be managed solely with systemic therapies with a palliative intent. However, the largest retrospective study on melanoma found that patients with progression of a single lesion during ICI had similar survival rates when local treatment was added to ICI, as local treatment, and ICI continuation alone [21].

SBRT represents one form of ablative localized treatment. It uses a non-invasive, high-precision radiotherapy technique characterized by its ability to deliver an inhomogeneous dose distribution. SBRT is administered in high doses over a few fractions and is associated with excellent local control [22]. Most studies of stereotactic radiation therapy in melanoma have focused on intracranial stereotactic radiosurgery (SRS) for which a benefit in survival and local control has been suggested, but there is a lack of evidence of the clinical benefit regarding SBRT for extracranial lesions [23, 24].

In this study we examine the outcome of SBRT in melanoma patients with OMD classified according to the ESTRO/EORTC criteria [9]. We report the local control, PFS, OS, and safety in the different OMD groups. Additionally, we analyze the prognostic factors for PFS and OS.

## Method

### Patients

All oligometastatic melanoma patients (*n* = 66) receiving SBRT in Stockholm, Sweden between 2010 and 2023 were included. OMD was defined as 1–5 metastases and the OMD status was further categorized according to the classification guidelines provided by ESTRO and EORTC [9]. Baseline characteristics were assessed at the timepoint of SBRT.

### Procedures

OMD was confirmed using computed tomography (CT) of the thorax and abdomen or an ^18^F-flourodeoxyglucose (FDG) positron emission tomography (PET)-CT scan prior to SBRT. All patients were discussed at a multidisciplinary tumor board to ascertain eligibility of SBRT. The prescription dose and fractionation schedules were selected according to local practice and based on tumor location, tumor size, and proximity to organs at risk (OAR). The stereotactic body frame, or a wing board in combination with a vacuum mold, was used for patient immobilization. Tumor motion during free breathing was analyzed using a 4D-CT scan, and abdominal compression was applied according to local protocols to reduce tumor motion. For margins, either the GTV (gross tumor volume)/CTV (clinical target volume)-PTV (planning target volume) or the ITV (internal target volume)-PTV-concept were used. Generally, with the CTV-PTV-concept, the PTV was created from the CTV using a margin of 5 mm in the transversal direction and 10 mm in the longitudinal direction, or greater if indicated by the amplitude of the tumor motion. If the ITV-PTV-concept was used, the ITV represented the position of the GTV throughout the breathing cycle and a margin of 4–5 mm was added around the ITV to create the PTV. The treatment was generally prescribed to about the 67% isodose line encompassing the PTV, which generated a maximum dose of approximately 150% of the prescribed dose in the center of the target. However, due to proximity to OAR or local routine, other isodose levels were also used for treatment prescription. Before each treatment fraction, a cone-beam CT was acquired to verify the target position and adjust the patient’s position as required. Patients were followed according to clinical routine, typically with CT or PET-CT every 3 months with additional evaluations performed as deemed necessary. Tumor evaluation was performed by a senior thoracic radiologist. Any records regarding radiotherapy related adverse events (AE) were collected and categorized according to Common Terminology Criteria for Adverse Events version 5.0 (CTCAE v5). Concurrent systemic therapy was defined as SBRT within 10 weeks of systemic treatment.

### Statistics

For descriptive data, median value was used for continuous variables, while counts and percentages were used for categorical variables. The chi-square test was used to analyze categorical variables representing patients’ baseline characteristics. Local control was calculated from the timepoint of SBRT to local failure or last follow up. PFS was measured from the timepoint of SBRT to progression of existing metastases, new metastatic lesions, death from any cause or last follow-up. OS was measured from the timepoint of SBRT until death from any cause or last follow-up. Time-to-event curves were performed using the Kaplan-Meier method and were compared between cohorts using log-rank statistics. The Cox proportional hazards model was used to determine significant prognostic factors of PFS and OS and to estimate hazard ratios (HRs) with associated 95% confidence intervals (CI). Model discrimination was assessed using the concordance probability estimate (CPE) by Gönen and Heller [25] with corresponding 95% CI. P-values < 0.05 were considered statistically significant. All statistical analyses were carried out using STATISTICA (Dell Inc., 2016), version 13.

## Results

### Baseline characteristics

A total of 66 patients with 72 metastatic lesions treated with SBRT were included in the analysis (Table 1). The median follow-up was 72.5 months (IQR 47.0-122.0 months). The median age was 68 years (range 39–90 years), and 42 (63.6%) patients were males. The majority (57.8%) exhibited a performance status of 0, while the remaining had ECOG 1–2 (42.2%). Sixty-two (93.9%) patients were diagnosed with cutaneous melanoma, while the remaining cases were diagnosed with mucosal melanoma. *BRAF* mutation was found in 23 (39.7%) patients. The distribution of M-stage among patients was as follows: 6 (9.1%) were classified as M1a, 30 (45.5%) as M1b, 18 (27.3%) as M1c, and 12 (18.2%) as M1d. The median number of metastases per patient was one (range 1–5). A total of 43 (65.2%) had a single metastasis, 12 (18.2%) had two metastases, and 11 (16.7%) had three to five metastases.

**Table 1:** Characteristics of melanoma patients with oligometastatic disease receiving stereotactic body radiation therapy

	Total *n* = 66 (%)	De novo *n* = 20 (%)	Repeat *n* = 25 (%)	Induced *n* = 21 (%)
Age, median (range)	68 (39–90)	64 (39–88)	67 (40–90)	69 (44–88)
Sex (%)				
Female	24 (36.4%)	8 (40.0%)	7 (28.0%)	9 (42.9%)
Male	42 (63.6%)	12 (60.0%)	18 (72.0%)	12 (57.1%)
ECOG performance status (%)				
0	37 (57.8%)	14 (70.0%)	11 (45.8%)	12 (60.0%)
1 & 2	27 (42.2%)	6 (30.0%)	13 (54.2%)	8 (40.0%)
NA	2		1	1
Type (%)				
Cutaneous	62 (93.9%)	19 (95.0%)	25 (100.0%)	18 (85.7%)
Mucosal	4 (6.1%)	1 (5.0%)	0 (0.0%)	3 (14.3%)
BRAF status (%)				
Wild type	35 (60.3%)	14 (73.7%)	10 (50.0%)	11 (57.9%)
V600 mutation	23 (39.7%)	5 (26.3%)	10 (50.0%)	8 (42.1%)
NA	8	1	5	2
M stage (%)				
M1a	6 (9.1%)	1 (5.0%)	3 (12.0%)	2 (9.5%)
M1b	30 (45.5%)	12 (60.0%)	14 (56.0%)	4 (19.0%)
M1c	18 (27.3%)	3 (15.0%)	3 (12.0%)	12 (57.1%)
M1d	12 (18.2%)	4 (20.0%)	5 (20.0%)	3 (14.3%)
Size of irradiated metastasis, median mm (range)	21.5 (8–68)	25 (10–68)	19 (10–65)	27 (9–58)
Number of metastases (%)				
1	43 (65.2%)	15 (75.0%)	14 (56.0%)	14 (66.7%)
2	12 (18.2%)	5 (25.0%)	5 (20.0%)	2 (9.5%)
3–5	11 (16.7%)	0 (0.0%)	6 (24.0%)	5 (23.8%)
RT to all metastatic lesions (%)				
Yes	46 (69.7%)	17 (85.0%)	16 (64.0%)	13 (61.9%)
No	20 (30.3%)	3 (15.0%)	9 (36.0%)	8 (38.1%)
Number of SBRT targets (%)				
1	61 (92.4%)	18 (90.0%)	22 (88.0%)	21 (100.0%)
2	4 (6.1%)	2 (10.0%)	2 (8.0%)	0 (0.0%)
3	1 (1.5%)	0 (0.0%)	1 (4.0%)	0 (0.0%)
RT target organ (%)				
Lung	44 (66.7%)	15 (75.0%)	18 (72.0%)	11 (52.4%)
^a^Soft tissue	10 (15.2%)	2 (10.0%)	4 (16.0%)	4 (19.0%)
^b^Abdominal organ	12 (18.2%)	3 (15.0%)	3 (12.0%)	6 (28.6%)
Prescribed dose in ^c^BED, median, Gy (range)	112.5 (56.0-112.5)	112.5 (72.0-112.5)	112.5 (56.0-112.5)	100.0 (59.5-112.5)
GTV mean dose in ^c^BED, median, Gy (range)	195.3 (59.5-262.5)	195.3 (89.5-205.8)	195.3 (85.3-205.8)	171.8 (59.5-262.5)
Staging imaging before SBRT (%)				
^d^CT thorax/abdomen	38 (57.6%)	10 (50.0%)	17 (68.0%)	11 (52.4%)
^e^PET/CT	28 (42.4%)	10 (50.0%)	8 (32.0%)	10 (47.6%)

^a^Soft tissue includes skin, lymph nodes and muscle

^b^Abdominal organ includes liver, adrenal gland and kidney

^c^Biological effective dose

^d^Computed tomography

^e^18-flourodeoxyglucose (FDG) positron emission tomography (PET)

### Classification of OMD

**Fig. 1:**
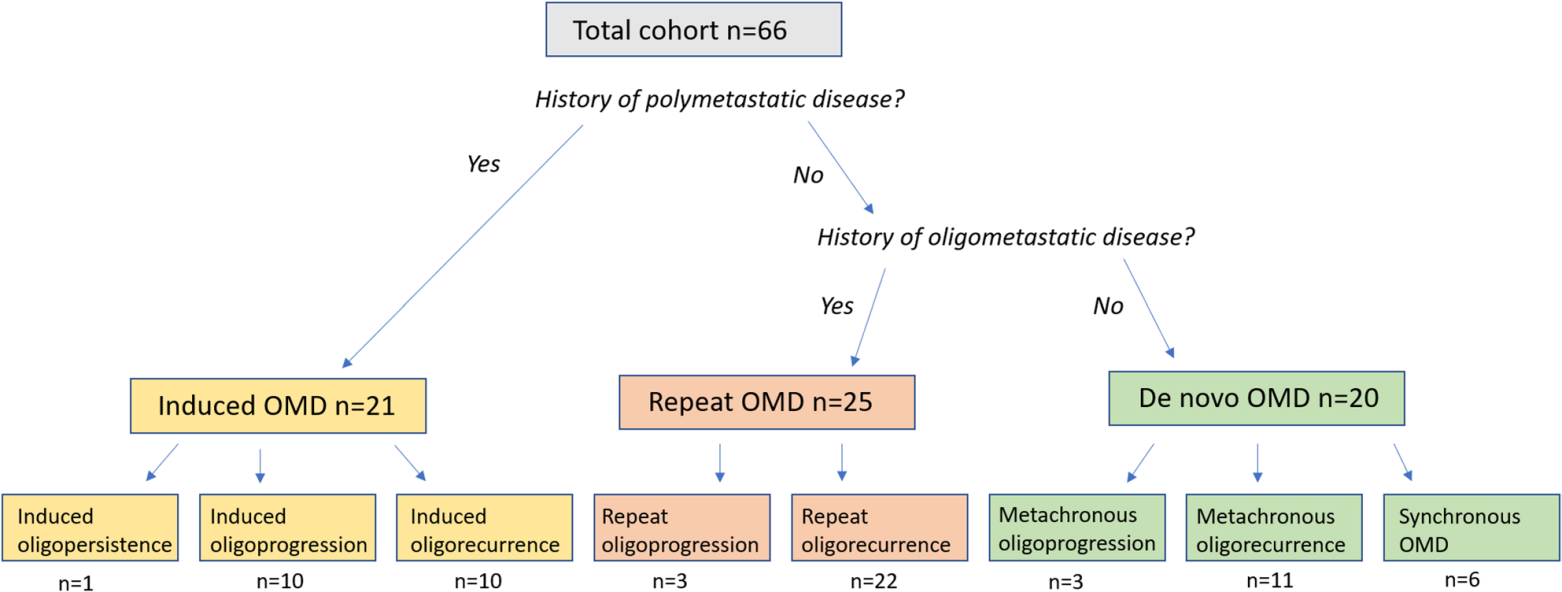
Distribution of oligometastatic disease (OMD) among melanoma patients treated with SBRT from 2010 to 2023, categorized based on the ESTRO/EORTC nomenclature

All patients were categorized at baseline according to ESTRO and EORTC’s classification of OMD, as having de novo (*n* = 20), repeat (*n* = 25), or induced (*n* = 21) OMD **(**Fig. 1**)**. The most prevalent OMD subcategories were repeat oligorecurrence, metachronous oligorecurrence, induced oligorecurrence and induced oligoprogression, accounting for 22 (33.3%), 11 (16.7%), 10 (15.2%), and 10 (15.2%) of cases, respectively. When comparing all baseline characteristics between the three OMD cohorts, M-stage was the only variable exhibiting statistically significant difference (*p* = 0.012) **(**Table 1**)**.

### SBRT treatment

The median size of the SBRT treated tumor at baseline was 21.5 mm (range 8–68 mm). SBRT was administered to all metastases in 46 (69.7%) patients. Most patients (92.4%) received SBRT targeting a single lesion, while the remaining five patients underwent SBRT targeting two or three lesions. The treated lesions were located either in the lungs (66.7%), skin, lymph nodes, muscles (soft tissues) (15.2%), or in the abdomen (18.2%). The median prescribed dose, fraction size, and number of fractions administered were 45 Gy (range 35–56 Gy), 15 Gy (range 4–15 Gy) and 3 fractions (range 3–10) respectively. The most frequent fractionation scheme was 15 Gy x3 (*n* = 39), followed by 10 Gy x5 (*n* = 12) and 8 Gy x5 (*n* = 5). The median of the minimum dose to the PTV was 38 Gy (range 18–49 Gy) and the median mean dose to the GTV was 64 Gy (range 35–78 Gy). Physical doses were converted to biological effective dose (BED) assuming an α/β ratio of 10 for tumor tissue (Supplementary Table 2).

### Systemic treatments

In the total study cohort, 25 patients (37.9%) had received prior systemic treatment, 20 patients (30.3%) received concurrent systemic treatment, and 38 patients (57.6%) received subsequent systemic treatment (Table 2). Among patients receiving systemic treatment, PD-1 inhibitor monotherapy was the most prescribed, accounting for 17 cases prior to SBRT, 8 cases concurrent with SBRT, and 15 cases post SBRT. Among patients treated with concurrent systemic therapy, the median time from SBRT to cessation of systemic therapy was 6.2 months (range 0.1–71.2 months). For those without concurrent systemic therapy, the median time until start of systemic therapy was 7.4 months (range 1.9-115.7) in patients who received subsequent treatment.

**Table 2:** Systemic treatments in oligometastatic melanoma patients treated with SBRT

	Total *n* = 66 (%)	De novo *n* = 20 (%)	Repeat *n* = 25 (%)	Induced *n* = 21 (%)
Systemic treatments prior SBRT				
Yes	25 (37.9%)	3 (15.0%)	6 (24.0%)	16 (76.2%)
No	41 (62.1%)	17 (85.0%)	19 (76.0%)	5 (23.8%)
Type of prior systemic treatment, n				
BRAF+/-MEKi	3	1	1	1
Anti PD-1	17	2	5	10
CTLA-4i	4	0	1	3
Anti PD-1 + CTLA-4i	3	0	1	2
Chemotherapy	4	0	0	4
Concurrent systemic treatment with SBRT				
Yes	20 (30.3%)	4 (20.0%)	5 (20.0%)	11 (52.4%)
No	46 (69.70%)	16 (80.0%)	20 (80.0%)	10 (47.6%)
Type of concurrent systemic treatment, n				
BRAF+/-MEKi	5	1	1	3
Anti PD-1	8	1	2	5
Anti PD-1 + CTLA-4i	3	1	1	1
Chemotherapy	4	1	1	2
Any prior or concurrent systemic treatment with SBRT				
Yes	38 (57.6%)	7 (35.0%)	10 (40.0%)	21 (100.0%)
No	28 (42.4%)	13 (65.0%)	15 (60.0%)	0 (0.0%)
Systemic treatment post SBRT				
Yes	38 (57.6%)	12 (60.0%)	14 (56.0%)	13 (61.9%)
No	28 (42.4%)	8 (40.0%)	11 (44.0%)	8 (38.1%)
Type of subsequent systemic treatment, n				
BRAF+/-MEKi	11	2	4	5
Anti PD-1	15	6	6	3
CTLA-4i	2	1	1	0
Anti PD-1 + CTLA-4i	9	3	5	1
Chemotherapy	10	3	4	3

### Local control

Local control rate at 1, 2, and 3 years was 96.3%, 93.2%, and 93.2%, respectively. Three local failures occurred at 5.1, 9.3, and 18.1 months, respectively. Two of the local failures were found in the induced OMD group, and one in the repeat OMD group. Local control was assessed in 63 of 72 lesions treated with SBRT. Nine tumors in seven patients were not evaluated radiologically post-SBRT, primarily due to rapid deterioration or death, and were therefore excluded from the analysis.

### Survival

**Fig. 2:**
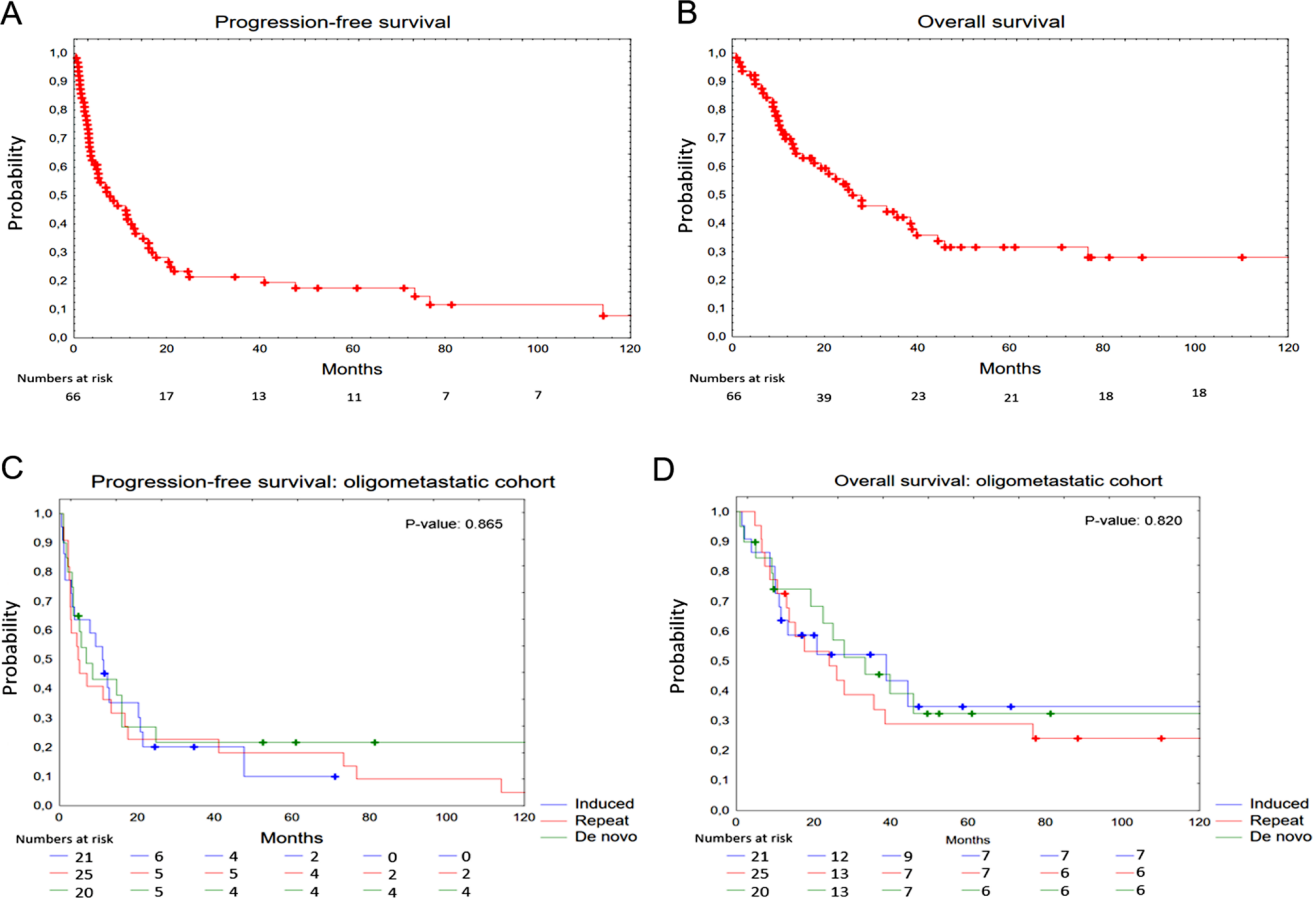
Kaplan-Meier curves illustrating survival in melanoma patients with oligometastatic disease (OMD) treated with SBRT. Panel **A** illustrates progression-free survival (PFS) in all melanoma patients with OMD treated with SBRT. Panel **B** depicts overall survival (OS) in all melanoma patients with OMD treated with SBRT. Panel **C** displays PFS based on the OMD cohorts established by ESTRO/EORTC: induced, repeat, and de novo. Panel **D** displays OS based on the OMD cohorts established by ESTRO/EORTC: induced, repeat, and de novo

The median PFS for the entire cohort (*n* = 66) was 7.7 (95% CI 4.9–12.4) months, while the median OS was 26.5 months (95% CI 17.6–38.8) (Fig. 2A and B). Median PFS in the de novo cohort was 6.6 months, 4.9 months in the repeat cohort, and 11.1 months in the induced cohort (Fig. 2C). The median OS was 29.2 months in the de novo cohort, 22.0 months in the repeat cohort, and 25.4 months in the induced cohort, with no significant differences observed in PFS (*p* = 0.865) or OS (*p* = 0.820) between the OMD cohorts (Fig. 2D). Additionally, no significant difference in OS was observed when comparing patients with oligoprogressive disease to those with other OMD subcategories (median OS 27.2 vs. 12.8 months) (*p* = 0.320), nor when comparing synchronous, metachronous, repeat and induced OMD (median OS 32.9 vs. 45.6 vs. 22.0 vs. 25.4 months) (*p* = 0.889).

**Fig. 3:**
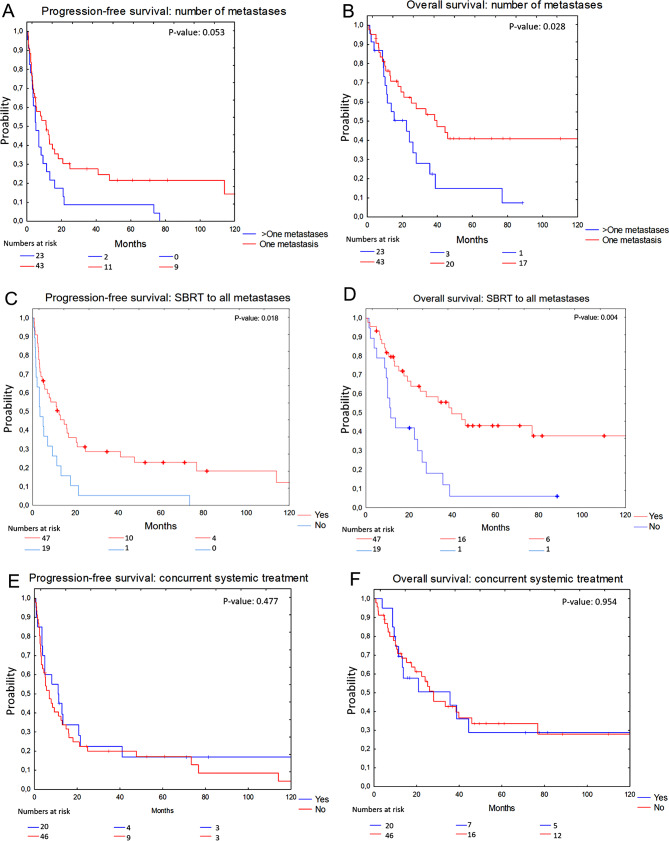
Kaplan Meier curves illustrating survival in melanoma patients with oligometastatic disease (OMD) treated with SBRT. Panel **A** displays progression-free survival (PFS) based on number of metastases (one/> one). Panel **B** displays overall survival (OS) based on the number of metastases (one/> one). Panel **C** illustrates PFS based on whether SBRT was administered to all known metastases (yes/no). Panel **D** demonstrated OS based on if SBRT was given towards all known metastases (yes/no). Panel **E** demonstrated PFS between patients treated with concurrent systemic treatment (yes/no). Panel **F** presents OS between patients treated with concurrent systemic treatment (yes/no)

However, improved PFS (median 11.4 vs. 5.2 months) and OS (median 39.6 vs. 14.9 months) were observed in patients with a single metastasis at baseline compared to those with multiple metastases (Fig. 3A and B). The difference was statistically significant for OS (*p* = 0.028) but not for PFS (*p* = 0.053). Furthermore, patients who received SBRT to all known metastatic lesions showed improved PFS (median 12.4 vs. 3.5 months) and OS (median 39.6 vs. 22.3 months) compared to those with remaining non-irradiated lesions (Fig. 3C and D) (*p* = 0.018 for PFS, *p* = 0.004 for OS). No significant difference in PFS (median 6.8 vs. 10.9 months) or OS (median 20.9 vs. 27.7 months) was seen if SBRT was administered with concurrent systemic treatment or not (Fig. 3E and F) (*p* = 0.477 for PFS, *p* = 0.954 for OS). When stratifying the survival analysis between OMD cohorts to include only patients treated with SBRT towards all known metastatic lesions, the results for PFS and OS showed no statistical significance (*p* = 0.078 for PFS, *p* = 0.137 for OS).

Univariable analysis (Table 3) demonstrated that age (≥ 65 years), ECOG performance status (1–2), number of metastatic lesions (2–5), and SBRT to all metastases (no) were associated with significantly worse OS. However, no statistically significant impact on OS was seen regarding sex, M-stage, *BRAF* status, baseline radiology, concurrent systemic treatment, size of irradiated tumor, target organ or OMD group (de novo, repeat, induced). Consistent with these findings, the ESTRO/EORTC OMD groups showed limited discriminative ability for PFS (CPE = 0.56 (95% CI 0.45–0.67)) and OS (CPE = 0.54 (95% CI 0.42–0.66)) (Supplementary Table 3). In the multivariable analysis, ECOG performance status (1–2) was the only variable associated with significantly worse OS after adjustment for age, concurrent systemic treatment, number of metastases, and SBRT to all metastases. Overall, the multivariable model demonstrated good discriminative ability in distinguishing patients with shorter versus longer PFS (CPE = 0.64 (95% CI, 0.56–0.71) and OS (CPE = 0.69 (95% CI, 0.62–0.77).

**Table 3:** Cox proportional hazard ratio for progression-free survival (PFS) and overall survival (OS) in oligometastatic melanoma treated with stereotactic body radiation therapy (SBRT)

	Univariate HR for PFS (95% CI)	*P*-value	Multivariate HR for PFS (95% CI)*	*P*-value	Univariate HR for OS (95% CI)	*P*-value	Multivariate HR for OS (95% CI)*	*P*-value
Sex								
Woman	REF				REF			
Man	0.99 (0.57–1.72)	0.975			0.75 (0.40–1.39)	0.356		
Age, years								
<65	REF		REF		REF		REF	
≥65	1.21 (0.71–2.08)	0.486	0.87 (0.47–1.62)	0.664	2.04 (1.06–3.89)	0.032	1.80 (0.89–3.66)	0.099
M stage								
1a-1b	REF				REF			
1c-1d	1.46 (0.86–2.47)	0.160			1.32 (0.71–2.45)	0.381		
BRAF status								
Wild type	REF				REF			
Mutated	1.15 (0.66–2.03)	0.620			0.86 (0.43–1.73)	0.675		
ECOG performance status								
0	REF		REF		REF		REF	
1–2	1.67 (1.07–2.60)	0.023	1.94 (1.17–3.22)	0.011	2.14 (1.14–4.1)	0.018	2.17 (1.10–4.30)	0.026
Baseline radiology								
CT	REF				REF			
PET/CT	0.80 (0.47–1.38)	0.426			0.64 (0.34–1.22)	0.175		
Concurrent systemic treatment								
Yes	REF		REF		REF		REF	
No	1.22 (0.68–2.18)	0.498	1.02 (0.54–1.91)	0.956	1.02 (0.52-2.00)	0.955	0.83 (0.41–1.70)	0.615
Size of irradiated tumor								
<22	REF				REF			
≥22	1.16 (0.68–1.97)	0.584			1.11 (0.60–2.06)	0.738		
Number of metastases								
1	REF		REF		REF		REF	
2–5	1.74 (1.01–2.98)	0.045	1.03 (0.35–2.98)	0.961	2.11 (1.12–3.97)	0.021	1.12 (0.33–3.86)	0.853
SBRT to all metastases								
Yes	REF		REF		REF		REF	
No	1.93 (1.10–3.41)	0.022	2.16 (0.69–6.73)	0.183	2.81 (1.48–5.36)	0.002	2.66 (0.71–10.01)	0.148
SBRT target								
Lung	REF				REF			
Other organs	0.90 (0.50–1.60)	0.712			0.85 (0.43–1.68)	0.643		
Oligometastatic cohort								
De novo	REF				REF			
Repeat	1.42 (0.74–2.71)	0.856			1.24 (0.58–2.62)	0.581		
Induced	1.07 (0.74–2.71)	0.290			1.27 (0.51–2.48)	0.767		

*Adjusting for age, ECOG performance status, number of metastases, if SBRT was given to all known metastases (yes/no) and concurrent systemic treatment (yes/no)

### Adverse events

Nineteen patients (28.8%) experienced grade 1–2 AEs attributable to SBRT. The most common AEs were cough (*n* = 4), skin erythema (*n* = 3), diarrhea (*n* = 3), pain (*n* = 3), and nausea (*n* = 3). No grade 3–5 AEs were observed. Similarly, among patients treated with concurrent ICIs (*n* = 11), three experienced AEs. Three out of five patients treated with concurrent BRAF/MEK inhibitors experienced AEs. In contrast, none of the four patients treated with concurrent chemotherapy reported AEs.

## Discussion

Our study suggests that SBRT in oligometastatic melanoma is associated with an excellent local control and a favorable toxicity profile. Interestingly, our analysis revealed no difference in PFS or OS among patients classified into the OMDs implemented by ESTRO/EORTC.

### OMD

In our study, the distribution of patients between the de novo (*n* = 20), repeat (*n* = 25) and induced (*n* = 21) cohorts were well balanced. The most prevalent OMD subtypes were repeat oligorecurrence and metachronous oligorecurrence. The “recurrence” subgroups refer to patients who developed OMD and received SBRT during a systemic therapy treatment-free interval. In such cases, the goal with SBRT is either to achieve stable disease if non-irradiated lesions are present, to acquire complete response if all metastases are treated, or to prolong the treatment-free interval of systemic therapy in order to preserve quality of life. Five additional studies have demonstrated that metachronous oligorecurrence is one of the most prevalent OMD subtypes [10, 11, 13–15]. However, one small study including NSCLC patients showed that induced oligopersistence and oligoprogression were the most common OMDs [12]. Although metachronous oligorecurrence is commonly reported, the distribution of OMD varies widely across studies, which include different primary tumors. The study most similar to ours in OMD distribution is a melanoma-specific study from France [15]. This suggest that metastatic patterns differ based on primary tumor histology. It could also indicate that SBRT use varies depending on the primary tumor type.

We defined OMD as 1–5 metastatic lesions, aligning with other SBRT trials [10, 20, 26]. However, the definition varies, with some limiting to 1–3 lesions [18, 27]. Additionally, certain studies include intracranial disease [10, 18, 20, 26], whereas others do not [27]. We advocate for future studies to adhere to a consensus definition of OMD, where ESTRO-ASTRO (American Society for Radiation Oncology) has suggested a maximum of 5 metastases, all suitable for SBRT. This standardization will aid in minimizing debate when comparing results across studies [28].

### Survival

In our total cohort, the median PFS was 7.7 months, and the median OS was 26.5 months. These results are substantially lower than those reported by a French retrospective study including oligometastatic melanoma patients treated with SBRT, which found a median PFS of 11.8 months and median OS of 86.5 months [15]. Despite similar OMD distribution and local control, survival differed significantly. This may be explained by their stricter inclusion criteria that required 1–3 metastases, ECOG < 2, and controlled intracranial disease. Furthermore, the before-mentioned study did not present the numbers of patients with brain metastases, a known poor prognostic factor, which limits direct comparison of survival outcomes.

Our survival outcomes align with a prospective UK registry study of 1442 patients treated with SBRT for extracranial oligometastases. Among melanoma patients (*n* = 58), a 2-year OS rate of 60.5% was reported [27]. Similarly, our results are comparable to those of Stinauer et al. [29], who reported a median OS of 22.2 for melanoma patients with OMD treated with SBRT. This is notable, given that their cohort included patients treated before the approval of targeted therapies and ICI. However, they defined OMD as 1–3 metastases in which all sites of disease were treated with local ablative treatment. Additionally, our results are consistent with those of the KEYNOTE-001 trial, which reported a median PFS of 8.3 months and a median OS of 23.8 months among metastatic melanoma patients treated with pembrolizumab; however, the cohort included both polymetastatic and OMD patients [30].

Our survival analysis showed no statistically significant differences in PFS or OS among the various OMDs implemented by ESTRO/EORTC, furthermore the OMD groups (de novo, repeat, and induced) showed limited discriminative ability for PFS and OS. Together, these findings suggest that the classification, when used in isolation, has a restricted capacity to distinguish between patients with shorter and longer survival. The numerically longer median OS in the de novo group, although not significant, may be attributable to their having undergone fewer prior systemic treatments, which could support better long-term outcomes. The lack of statistically significant separation between OMD groups contrast with findings from five validation studies of the ESTRO/EORTC nomenclature which have demonstrated a difference [9]. For instance, Willmann et al. demonstrated in a mixed histology setting, that the median PFS in de-novo cohort (8.8 months) was significantly longer than in repeat (5.4 months) and induced OMD (4.3 months). Additionally, they observed shorter OS among the induced OMD compared to both de novo and repeat OMD [10]. A study including NSCLC patients with OMD treated with SBRT, demonstrated that induced oligoprogression was associated with worse OS compared to patients with non-induced oligoprogressive disease [12], another study with mixed primary tumors showed that oligoprogression had the least favorable PFS [11]. Three studies demonstrating better survival outcomes among those with synchronous OMD, two of them involved prostate cancer [13, 14] and the third study included mixed primaries [11]. The single study indicating no significant survival difference between OMDs is a French retrospective melanoma study [15]. This, in conjunction with our findings, suggest that while the ESTRO/EORTC classification effectively categorizes oligometastatic melanoma patients, its prognostic value may be limited. Instead, it may primarily function as a standardized communication tool. Due to our cohort size which limits statistical power, we look forward to results of larger prospective trials exploring the prognostic value in melanoma.

OMD diagnosis is based on imaging and no biomarker exists [9]. It is natural to assume that patients evaluated by advanced radiology would experience improved survival, since this imaging technology makes it easier to distinguish between OMD and widespread disease. However, we could not find a significant difference in PFS or OS when comparing patients examined with PET/CT before SBRT with those examined with CT.

Most trials studying OMD patients receiving SBRT have typically treated all metastases. However, in our study cohort, 30.3% of patients had remaining non-irradiated lesions. We observed better survival among patients who received SBRT to all known metastases. However, this finding was not significant after adjusting for age, ECOG performance status, number of metastatic sites, and concurrent systemic treatment. These findings present a challenge in interpretation. It remains unclear whether they support the definition proposed by the ASTRO and ESTRO consensus or not, which suggests that all metastatic sites should be treated [28].

### Local control

Our study demonstrated excellent local control rates of 96.3%, 93.2%, and 93,2% at 1, 2, and 3 years, respectively. These outcomes surpass those reported by Franceschini et al.t, who observed local control rates of 96.6%, and 82.8% at 1 and 2 years, respectively [31], and by Stinauer et al., who reported a 1 year local control rate of 82% [29]. This is notable since a higher proportion of patients in the Franceschini study had a higher proportion of patients with a prescribed BED > 100 Gy compared to our study (74% vs. 56%). However, these two studies were conducted earlier, before the widespread establishment of modern systemic treatments that can potentially enhance the efficacy of radiotherapy [32]. It is worth noting that our results are also superior to a large registry study including mixed histologies. Among the melanoma patients included, the reported local control rate at 1 and 2 years was 81.2% and 67.7% respectively [27]. However, our results closely align with a melanoma-specific study, which reported local control rates of 94.2%, 90.3%, and 90.3% at 1, 2, and 3 years respectively [15]. This similarity can be attributed to comparable study populations, the timing of the studies and similar dosimetry data. Notably, 68% of patients in their study received concurrent systemic treatments, compared to 30.3% in ours. Despite this, both studies reported similar local control, which challenges the theory that concurrent systemic therapy enhances local control [32].

### Adverse events

Consistent with published studies [20, 27, 29, 31, 33], we demonstrated that SBRT can be safely administered, with no reported grade 3–5 AEs. Among the entire cohort, only 19 (28.7%) patients experienced grade 1–2 AEs attributable to SBRT.

### Strengths and limitations

This study stands out as one of the first to specifically focus on oligometastatic melanoma, providing valuable insights into the survival outcomes, local response, and safety of SBRT within the OMDs definitions established by ESTRO/EORTC. By addressing this relatively underexplored area, our research contributes significantly to the growing body of evidence supporting SBRT as a promising treatment option for melanoma patients with OMD. The main limitations of our study is its retrospective design, the single-center nature, and relatively small cohort size. The small cohort size limits statistical power but also reflect the limited integration of SBRT into clinical practice.

## Conclusion

We demonstrated that SBRT for oligometastatic melanoma is associated with excellent local control and a favorable toxicity profile. Our analysis could not identify a specific OMD category, as classified by ESTRO/EORTC nomenclature, that derived the greatest benefit from SBRT. These results suggest that the current ESTRO/EORTC nomenclature may have limited prognostic utility in oligometastatic melanoma. Further large-scale, prospective trials are warranted to more precisely determine which melanoma patients with OMD are most likely to benefit from local ablative therapies.

## Supplementary Information

Below is the link to the electronic supplementary material.

Supplementary Material 1

## Data Availability

The data that support the findings of this study are available from the corresponding author E.H and specific data will be available on reasonable request.

## Abbreviations

AE: Adverse Events
ASTRO: American Society for Radiation Oncology
CI: Confidence intervals
CT: Computed tomography
CTCAE: Common Terminology Criteria for Adverse Events
CTV: Clinical target volume
ECOG: Eastern Cooperative Oncology Group
EORTC: European Organization for Research and Treatment of Cancer
ESTRO: European Society for Radiotherapy and Oncology
FDG: ^18^F-flourodeoxyglucose
GTV: Gross tumor volume
HR: Hazard ratios
ICI: Immune checkpoint inhibitors
ITV: Internal target volume
LDH: Lactate dehydrogenase
OMD: Oligometastatic disease
NSCLC: Non-small cell lung cancer
OAR: Organs at risk
OS: Overall survival
PET: Positron emission tomography
PFS: Progression-free survival
PTV: Planning target volume
SBRT: Stereotactic body radiation therapy
SRS: Stereotactic radiosurgery
