# Supplementary material for: Stereotactic body radiation therapy for oligometastatic melanoma: a real-world study of the ESTRO/EORTC nomenclature
Source: Radiat Oncol. 2026 Jan 30;21:39. doi: 10.1186/s13014-026-02792-2 (PMC12951983; doi:10.1186/s13014-026-02792-2)
Supplement: Supplementary file 1 — Supplementary Material 1 [file 13014_2026_2792_MOESM1_ESM.docx]

**Supplementary Material**


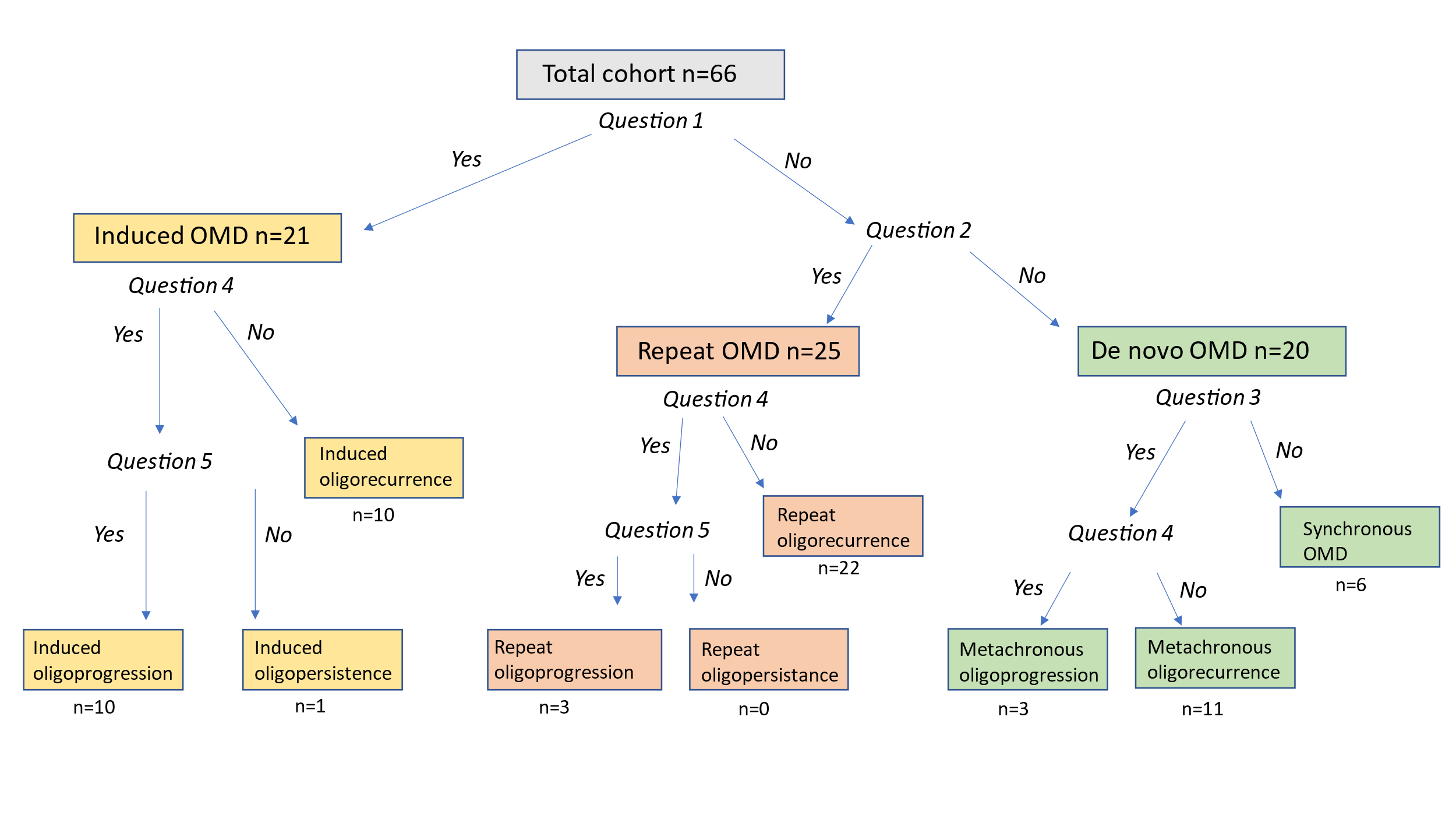


**Supplementary Figure 1*.*** Decision tree for the classification of oligometastatic disease according to Guckenberger et al, (2020)

| Question 1: Does the patient have a history of polymetastatic disease before current diagnosis of oligometastatic disease? |
| --- |
| Question 2: Does the patient have a history of oligometastatic disease before the current diagnosis of oligometastatic disease? |
| Question 3: Has oligometastatic disease been first diagnosed more than 6 months after the primary cancer diagnosis? |
| Question 4: Is the patient under active systemic therapy at the time of oligometastatic disease diagnosis? |
| Question 5: Are any oligometastatic lesions progressive on current imaging? |

| **Supplementary Table 1. Distribution of oligometastatic subgroups in melanoma patients receiving SBRT** | | | | |
| --- | --- | --- | --- | --- |
| Oligometastatic setting | Total cohort (n=66) | De novo (n=20) | Repeat (n=25) | Induced (n=21) |
| ^a^Synchronous oligometastatic disease | 6 (9.1%) | 6 (30.0%) | 0 (0%) | 0 (0%) |
| ^b^Metachronous oligoprogression | 3 (4.5%) | 3 (15.0%) | 0 (0%) | 0 (0%) |
| ^c^Metachronous oligorecurrence | 11 (16.7%) | 11 (55.0%) | 0 (0%) | 0 (0%) |
| ^d^Repeat oligoprogression | 3 (4.5%) | 0 (0%) | 3 (12%) | 0 (0%) |
| ^e^Repeat oligorecurrence | 22 (33.3%) | 0 (0%) | 22 (88%) | 0 (0%) |
| ^f^Repeat oligopersistance | 0 (0%) | 0 (0%) | 0 (0%) | 0 (0%) |
| ^g^Induced oligopersistence | 1 (1.5%) | 0 (0%) | 0 (0%) | 1 (4.5%) |
| ^h^Induced oligoprogression | 10 (15.2%) | 0 (0%) | 0 (0%) | 10 (47.6%) |
| ^i^Induced oligorecurrence | 10 (15.2%) | 0 (0%) | 0 (0%) | 10 (47.6%) |

| ^a^First time diagnosis of oligometastatic disease with less than 6 months elapsed since primary diagnosis. |
| --- |
| ^b^First-time diagnosis of oligometastatic disease with more than 6 months elapsed since primary diagnosis, currently undergoing systemic therapy . |
| ^c^First-time diagnosis of oligometastatic disease, with more than 6months elapsed since primary diagnosis, and is currently not receiving active systemic therapy. |
| ^d^Previous history of oligometastatic disease, and is on active systemic therapy with at least one progressing tumor. |
| ^e^Previous history of oligometastatic disease and is currently not receiving active systemic therapy  ^f^Previous history of oligometastatic disease, and is on active systemic therapy with no progressing tumors. |
| ^g^A history of polymetastatic disease that has transitioned to an oligometastatic state and is currently on active systemic therapy with no evidence of tumor progression. |
| ^h^A history of polymetastatic disease that has transitioned to an oligometastatic state and is currently on active systemic therapy with at least one progressing tumor. |
| ^i^A history of polymetastatic disease that has transitioned to an oligometastatic state and is currently not receiving active systemic therapy |
|  |

| **Supplementary table 2. Fractionation scheme of all metastases treated with SBRT** | | | | | |
| --- | --- | --- | --- | --- | --- |
| Total dose (Gy) | Fractionation scheme | EQD2 (α/β =10) | GTV mean physical dose, Gy: median (range) | PTV minimum physical dose, Gy: median (range) | n (%) |
| 40 | 4 Gy x10 | 47 | 58 (58-58) | 32 (32-32) | 1 (1.4) |
| 40 | 5 Gy x8 | 50 | 58 (58-58) | 18 (18-18) | 1 (1.4) |
| 50 | 5 Gy x10 | 63 | 63 (55-72) | 37 (29-42) | 4 (5.6) |
| 48 | 6 Gy x8 | 64 | 69 (69-69) | 34 (34-34) | 1 (1.4) |
| 52 | 6.5 Gy x8 | 72 | 70 (70-70) | 18 (18-18) | 1 (1.4) |
| 35 | 7 Gy x5 | 50 | 35 (35-35) | 30 (30-30) | 1 (1.4) |
| 56 | 7 Gy x8 | 79 | 74 (71-78) | 32 (28-46) | 3 (4.2) |
| 40 | 8 Gy x5 | 60 | 58 (47-59) | 37 (28-39) | 5 (6.9) |
| 45 | 9 Gy x5 | 71 | 58 (51-65) | 39 (38-40) | 2 (2.8) |
| 50 | 10 Gy x5 | 83 | 71 (55-73) | 39 (22-49) | 12 (16.7) |
| 45 | 15 Gy x3 | 94 | 64 (62-75) | 40 (19-46) | 41 (56.9) |

| **Supplementary Table 3. Concordance probability estimate (CPE) for progression-free survival (PFS) and overall survival (OS) in oligometastatic melanoma treated with stereotactic body radiation therapy (SBRT)** | | |
| --- | --- | --- |
| Variable | CPE PFS (95% CI) | CPE OS (95% CI) |
| Sex (female vs. male) | 0.50 (0.36-0.64) | 0.57 (0.42-0.73) |
| Age (<65 vs. ≥65) | 0.55 (0.41-0.68) | 0.67 (0.53-0.82) |
| M stage (1ab vs. 1cd) | 0.59 (0.47-0.72) | 0.56 (0.41-0.71) |
| BRAF status (wild type vs. mutated) | 0.54 (0.40-0.68) | 0.55 (0.37-0.72) |
| ECOG performance status (0 vs. 1-2) | 0.64 (0.53-0.75) | 0.72 (0.61-0.84) |
| Baseline radiology (CT vs. PET/CT) | 0.55 (0.42-0.69) | 0.61 (0.46-0.76) |
| Concurrent systemic treatment (yes vs. No) | 0.55 (0.41-0.69) | 0.50 (0.34-0.67) |
| Size of irradiated tumor (<22mm vs. ≥22mm) | 0.54 (0.41-0.67) | 0.53 (0.37-0.68) |
| Number of metastases (1 vs. 2-5) | 0.63 (0.51-0.76) | 0.68 (0.54-0.82) |
| SBRT to all metastases (yes vs. no) | 0.66 (0.53-0.79) | 0.69 (0.55-0.83) |
| SBRT target (lung vs. other organs) | 0.53 (0.38-0.67) | 0.54 (0.37-0.71) |
| Oligometastatic cohort (de novo vs. repeat vs. induced) | 0.56 (0.45-0.67) | 0.54 (0.42-0.66) |
| **Multivariable model*** (long vs. short PFS/OS) | 0.64 (0.56-0.71) | 0.69 (0.62-0.77) |

*The multivariable model includes age, ECOG performance status, number of metastases, if SBRT was given to all metastases, and concurrent systemic treatment.
